# Reversible Cortical Blindness as a Prominent Manifestation of Cerebral Embolism due to Infective Endocarditis

**DOI:** 10.1155/2010/408471

**Published:** 2010-08-09

**Authors:** Georgios P. Kranidiotis, Alexandra N. Gougoutsi, Theodoros A. Retsas, Maria I. Anastasiou-Nana

**Affiliations:** Department of Clinical Therapeutics, School of Medicine, University of Athens, “Alexandra” Hospital, 80 Vasilissis Sofias Av. 115 28 Athens, Greece

## Abstract

*Introduction*. Infective endocarditis in the left heart may be complicated by stroke, due to embolisation from infectious valvular vegetations. Infarction of both occipital lobes, which are supplied by the posterior cerebral arteries, is infrequent, and is the cause of cortical blindness from lesion of the visual cortex. Cortical blindness is characterized by intact pupillary reflexes, a normal fundoscopy, and, rarely, denial of visual loss. *Case Presentation*. We report the case of a 58-year-old woman, recipient of a mechanical aortic valve, who presented with fever, multiple organ dysfunction, and cortical blindness. Transesophageal echocardiography and blood cultures confirmed the diagnosis of infective endocarditis caused by methicillin-sensitive *Staphylococcus aureus*. Computed tomography of the brain without contrast revealed the presence of infarctions in both occipital lobes. It is noteworthy that the visual loss resolved after treatment of endocarditis. *Conclusions*. A stroke occurring in a patient presenting with fever and a history of valvular heart disease strongly suggests the presence of infective endocarditis. Bilateral thromboembolic infarcts of the occipital lobes cause cortical blindness, that can resolve after treatment of endocarditis.

## 1. Introduction

Patients presenting with infective endocarditis suffer from a 25 to 35% incidence of cerebrovascular complications due to embolisation from endocardial vegetations and occlusions of cerebral arteries. Most cerebrovascular complications are apparent on admission to the hospital or develop shortly thereafter. Thus, an ischemic stroke may be the initial clinical manifestation of endocarditis [1, 2].

Cortical blindness is the total or partial loss of vision in presence of apparently normal ocular examination and fundoscopy, caused by bilateral lesions of the primary visual cortex in the occipital lobe. Despite the loss of vision, the pupillary reflex to light is intact, as it does not involve the cortex. Anton has described a rare form of cortical blindness denied by the patient, despite complete visual loss (Anton's syndrome) [3]. Bilateral occlusion of the posterior cerebral arteries, which supply the occipital lobes, may be the cause of cortical blindness [4]. 

We describe a patient presenting with an aortic prosthetic valve, fever and cortical blindness, which resolved after treatment of endocarditis.

## 2. Case Presentation

A 58-year-old woman, recipient of a mechanical aortic valve, suddenly lost vision 1 hour after being admitted to the hospital for evaluation of a 2-day history of fever up to 39.5°C. Although she was aware of being blind, she underestimated the seriousness of her condition and seemed unconcerned. The patient, who had undergone replacement of a stenotic aortic valve 7 years earlier, had a history of arterial hypertension and dyslipidaemia. Her family history was negative for valvular heart disease, rheumatismal diseases, stroke, or other neurologic disorders. The patient was a widow, retired accountant, living alone in Athens. She had not travelled outside the country and had not been exposed to contagious illnesses. She had never smoked and did not consume alcohol or use illegal drugs. Her daily medication regimen included acenocoumarol, metoprolol, and lisinopril. She had a history of allergy to *β*-lactam antimicrobials. 

On physical examination, the patient appeared critically ill and had faintly icteric skin and sclerae. Her temperature was 37°C, blood pressure 100/60 mmHg, and heart rate 90 bpm. The second heart sound was consistent with a mechanical prosthetic valve. The pupillary responses to light were intact and the fundoscopy was normal. The remainder of the examination was normal. 

A complete blood count showed 13,000 leukocytes/mm^3^, 97% granulocytes, and 2% lymphocytes. The hemoglobin was 14 g/dL, platelets 135,000/mm^3^, international normalized ratio 4.3, erythrocyte sedimentation rate 125 mm/h, C-reactive protein 38 mg/dL (normal < 0.5 mg/dL), fibrinogen 7.9 g/L, serum creatinine 3.2 mg/dL, blood urea nitrogen 82 mg/dL, aspartate aminotransferase 88 U/L, alanine aminotransferase 104 U/L, *γ*-glutamyltransferase 477 U/L, alkaline phosphatase 205 U/L, total serum bilirubin 5.2 mg/dL, direct bilirubin 3.5 mg/dL, and lactate dehydrogenase 430 U/L. The urine analysis revealed 2+ protein, and 5 to 8 erythrocytes, and 15 to 20 leukocytes per high-power field. The urinary sediment contained tubulo-epithelial cells and muddy-brown granular casts, no dysmorphic erythrocytes and no red-cell casts. The tests for hepatitis B surface antigen, hepatitis B core IgM antibody, hepatitis A IgM antibody, and hepatitis C antibody were negative. While the urine was sterile, 3 sets of blood cultures obtained at the time of admission grew methicillin-sensitive *Staphylococcus aureus*.

**Figure 1:**
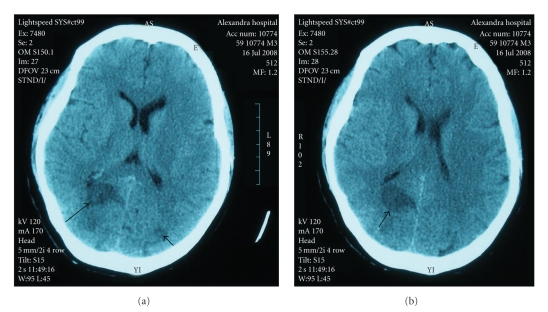
Computed tomography of the brain without contrast, obtained 24 hours after admission of the patient to the hospital: infarcts are present in both occipital lobes (arrows).

**Figure 2:**
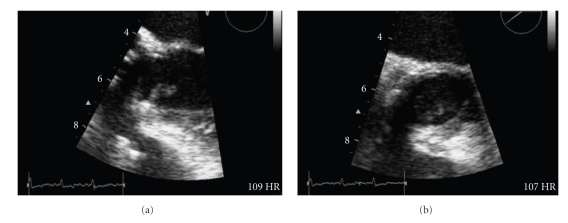
Transesophageal echocardiogram showing a pedunculated vegetation attached to the prosthetic aortic valve. (a) 0° horizontal midesophageal view. (b) 34° midesophageal short cardiac axis.

A few hours later, the patient became hypotensive and was admitted to the intensive care unit. Treatment with intravenous fluids, gentamicin, and vancomycin was begun, and anticoagulation was discontinued. A first computed tomography scan of the brain without contrast, obtained at the time of admission, was negative. A follow-up scan, performed 24 hours later, showed infarctions in both occipital lobes (Figure 1). A transeosophageal echocardiogram showed the presence of a vegetation on the prosthetic aortic valve (Figures 2(a) and 2(b)). Also see Videos 1 and 2 in Supplementary Material available online at doi: 10.1155/2010/408471.

Over the following 4 days, the patient became hemodynamically stable and her renal and liver function tests returned toward normal values. On the 20th hospital day, she was afebrile, and her vision was partially restored. On transesophageal echocardiographic examination, 1 month after admission, the vegetation had disappeared, and she suffered no further clinical manifestations of systemic embolisation.

## 3. Discussion

Any unexplained illness in a patient with a history of underlying cardiac valvular disease should raise the suspicion of infective endocarditis. In our patient, fever associated with multiple organs dysfunction was consistent with a systemic infection. Positive blood cultures and vegetations detected on the echocardiogram, 2 major Duke diagnostic criteria, confirmed the diagnosis of infective endocarditis [5].

An unusual observation, in this case, was the location of the septic cerebral infarct. The cerebral territory most commonly affected by infective endocarditis-related embolism is that supplied by the middle cerebral artery, whereas infarcts in the territory of the posterior cerebral arteries are infrequent [6]. *Staphylococcus aureus*, the pathogen isolated from our patient, is associated with a higher risk of cerebrovascular complications than other bacteria [7]. 

In patients presenting with infective endocarditis, an early diagnosis and initiation of antimicrobial therapy is of major importance in the prevention of embolic stroke. The probability of developing this complication decreases rapidly after the initiation of antimicrobial therapy, presumably owing to the stabilization of the vegetations [2]. We empirically chose a regimen of vancomycin and gentamicin immediately after the collection of blood cultures, to cover the common bacteria causing endocarditis >12 months after prosthetic valve implantation, including *Streptococcus *species, *Staphylococcus aureus*, coagulase-negative staphylococci, *Enterococcus* species, and gram-negative bacilli. The decision to initiate treatment before the confirmation of endocarditis was prompted by the deteriorating hemodynamic status and neurologic complications. Rifampin was withheld because of abnormal liver function tests. Although blood cultures grew methicillin-sensitive *Staphylococcus aureus*, we did not substitute a *β*-lactam for vancomycin because of her history of allergy.

In patients presenting with prosthetic valve endocarditis due to *Staphylococcus aureus* who have suffered a recent cerebrovascular embolism, anticoagulation must be stopped for ≥ 2 weeks after the onset of antimicrobial therapy to allow organization of the thrombus and prevent the development of acute hemorrhagic complications [8]. As generally recommended, we reintroduced anticoagulation 2 weeks after the patient's admission to the hospital.

## 4. Conclusions

A stroke occurring in a patient presenting with fever and a history of valvular heart disease strongly suggests the presence of infective endocarditis. Bilateral thromboembolic infarcts of the posterior cerebral arteries are rare causes of lesions of the occipital lobes and cortical blindness, that is loss of vision with intact pupillary reflexes and normal fundoscopy.

## Supplementary Material

Video 1: Transesophageal Echocardiogram. A 0° horizontal midesophageal view from a transesophageal echocardiogram performed on the 2nd hospital day. A pedunculated vegetation is attached to the prosthetic aortic valve.
Video 2: Transesophageal Echocardiogram. A 34° midesophageal short cardiac axis view from a transesophageal echocardiogram performed on the 2nd hospital day. A pedunculated vegetation is attached to the prosthetic aortic valve.
